# Kinematic alignment in Japanese patients shows significant improvement 2 years after total knee replacement surgery

**DOI:** 10.1002/jeo2.70264

**Published:** 2025-05-12

**Authors:** Takao Yamamoto, Shigenobu Fukushima, Shuji Toyono, Takahiro Miyaji, Taku Nakajima, Yoshihiro Wanezaki, Takashi Ito

**Affiliations:** ^1^ Department of Orthopedic Surgery Yamagata Prefectural Shinjo Hospital Shinjo‐shi Yamagata Japan; ^2^ Artificial Joint Center Yamagata Saisei Hospital Yamagata Japan; ^3^ Department of Orthopedic Surgery Public Okitama General Hospital Okitama Yamagata Japan; ^4^ Department of Orthopedic Surgery Yamagata University Faculty of Medicine Yamagata‐shi Yamagata Japan

**Keywords:** cinical evaluations, Japanese patients, kinematic alignment, radiographic measurements, total knee arthroplasty, two‐year outcomes

## Abstract

**Purpose:**

This study aimed to evaluate the 2‐year post‐operative results of the unrestricted kinematic alignment technique in total knee arthroplasty (TKA) among Japanese patients and understand the potential benefits of this technique.

**Methods:**

In total, 207 patients who underwent kinematic alignment in TKA for knee osteoarthritis between 2019 and 2021 were retrospectively reviewed. After applying the exclusion criteria, 164 knees remained for analysis (130 and 34 knees from female and male patients, respectively). The average age and body mass index were 74.5 ± 8.0 years and 26.4 ± 4.1 kg/m^2^, respectively. Radiographic measurements were conducted preoperatively and post‐operatively, while clinical evaluations—including knee extension, flexion angles, muscle strength, gait patterns and Knee Society scores (patient satisfaction and function)—were performed preoperatively, 1 year post‐operatively and 2 years post‐operatively. Statistical analysis was used to determine interobserver reliability and compare the preoperative and post‐operative values. Radiographic evaluations were analyzed using a paired Student's *t* test, while clinical evaluations were analyzed using one‐way ANOVA followed by a Tukey–Kramer multiple comparison test. Statistical significance was set at *p* < 0.05.

**Results:**

After surgery, statistically significant improvements were observed in both knee extension and flexion angles, as well as muscle strength (*p* < 0.01). Two years after surgery, the rates of independent indoor walking and stair climbing were 89.6% and 58.9%, respectively. Additionally, the Knee Society Scores (patient satisfaction and function) significantly improved compared with the preoperative status (*p* < 0.01). Complications were minimal; revision surgery was not required.

**Conclusion:**

In Japanese patients, kinematic alignment in TKA demonstrated significant improvements and promising outcomes over 2 years. Although alignment characteristics prior to arthritis may vary due to ethnic differences, this method—designed to replicate patient‐specific alignments—is considered to have achieved favourable outcomes by tailoring to individual alignments. Further research comparing kinematic alignment with conventional alignment techniques could provide more valuable insights.

**Level of Evidence:**

Level III.

**Abbreviations**
FJS‐12: Forgotten Joint Score
HKA: hip–knee–ankle angle
ICC: intraclass correlation coefficient
KA‐TKA: kinematic alignment in TKA
KSS: Knee Society Score
MA: mechanical alignment
mLDFA: mechanical lateral distal femoral angle
MPTA: medial proximal tibial angle
TKA: total knee arthroplasty

## INTRODUCTION

Total knee arthroplasty (TKA) is a common surgical procedure for treating knee osteoarthritis. Although conventional mechanical alignment (MA) is widely used [1, 13, 16], more individualized approaches to alignment have been explored in recent years. These methods aim to achieve better soft tissue balance by placing the implant closer to the constitutional alignment than in the MA method [4, 5, 21, 28, 29]; among these, kinematic alignment in TKA (KA‐TKA) is considered a surgical technique that restores the surfaces of the femur and tibia to their original articular surfaces and reproduces the patient's unique joint line, thus achieving more patient‐specific alignment and ligament balance [7, 15, 26]. Ethnic differences result in variations in pre‐arthritic alignment. It has been reported that in Japanese individuals, the mechanical lateral distal femoral angle (mLDFA; the between the femoral mechanical axis and distal articular surface) and medial proximal tibial angle (MPTA; angle between the tibial mechanical axis and tibial articular surface, or base plate of the tibial component) are smaller, while the hip–knee–ankle angle (HKA; angle between the femoral mechanical axis and tibial functional axis [positive is varus]) is larger; moreover, the percentage of constitutional varus is higher in Japan compared to that of other countries [33]. Given that KA aims to replicate the patient's individual articular surface, it is anticipated that post‐operative alignments following KA‐TKA may also differ among ethnicities. However, studies on the outcomes of KA‐TKA in Japanese patients are limited [17, 25]. Therefore, this study aimed to investigate the 2‐year post‐operative outcomes of unrestricted KA‐TKA in Japanese patients.

## MATERIALS AND METHODS

### Patients

This retrospective study enroled 207 consecutive patients who underwent KA‐TKA for knee osteoarthritis at a single institution between November 2019 and September 2021. The exclusion criteria were extraarticular deformities resulting from trauma, osteotomy or post‐operative THA, and missing measurement data. The excluded cases comprised 4 knees with extraarticular deformities due to previous trauma, 1 knee after osteotomy, 1 knee after THA and 37 knees with missing measurement data; thus, 164 knees (130 female and 34 male) were included in the final analysis. The mean age of the patients included in the final analysis was 74.5 ± 8.0 years (range: 44–91), while the mean body mass index was 26.4 ± 4.1 kg/m^2^ (range: 17.5–39.7).

### Surgical procedure

The surgical technique involved unrestricted KA‐TKA with a mid‐vastus approach. Osteotomy thickness was measured intraoperatively according to the method reported by Howell et al. [15]. After correction for cartilage wear and bone identified thickness in the distal and posterior femurs, osteotomy thicknesses of the distal and posterior femurs were adjusted to within 0 ± 0.5 mm of the component thickness. In the original method by Howell et al. [15], the tibia was measured by determining the thickness of the component originating from the tibial articular surface; however, due to the challenges in assessing the tibial articular surface in Japanese patients, which are often attributed to prevalent severe varus deformities and bone loss, we consulted the manual inline traction technique proposed by Brown [6]. This is a soft‐tissue‐respecting technique wherein the tibial joint plane is aligned parallel to the femoral osteotomy plane, and the tibial osteotomy angle is checked while the lower leg is extended [12].

The posterior tibial tilt was adjusted by confirming the tibial tilt with the plane of the ‘angel wing’ inserted into the tibial guide in relation to the medial articular plane. Balance after osteotomy was adjusted using an additional tibial osteotomy [15]. All the implants were fixed with cement. The implant was a GMK Sphere® (Medacta). All surgeries were performed by one of two surgeons (SF and TY).

### Radiographic examination

#### Radiographic method

Radiographs of the full‐length lower extremities were taken preoperatively and post‐operatively in a standing position, with the cassette placed 300 cm from the source (settings: 85 kV and 200 mA) [11]. The radiographs were captured with both legs positioned close together. Image analysis was performed using EV Insite (version 3; PSP Corporation).

#### Radiographic measurements

The radiographic alignment parameters included the (a) HKA (angle between the femoral mechanical axis and tibial functional axis [positive is varus]); (b) mLDFA (angle between the femoral mechanical axis and distal articular surface); and (c) MPTA (angle between the tibial mechanical axis and tibial articular surface, or base plate of the tibial component). All radiographic alignment parameters were assessed by two observers (TY and ST).

### Clinical evaluation

All patients were examined preoperatively and 1 and 2 years post‐operatively by a physical therapist. Knee extension and flexion angles were measured using a long‐arm goniometer. Knee extension muscle strength was measured using a handheld dynamometer (μ‐Tas FI; Anima Corporation) with the knee joint at 60° flexion while seated. Gait patterns were assessed during indoor walking while ascending and descending stairs. Furthermore, patient satisfaction and function (scored from 0 to 40 and 0 to 100 [*worst* to *best*], respectively, using the Knee Society Score [KSS] [30]) were evaluated preoperatively and 1 and 2 years post‐operatively. The 12‐point Forgotten Joint Score (FJS‐12) [2, 10], scored from 0 to 100 (*worst* to *best*), was also assessed 1 and 2 years post‐operatively. A retrospective evaluation was conducted to determine the presence of complications requiring revision.

### Statistical analysis

All statistical analyses were performed using GraphPad Prism version 10.0.3 for Windows (GraphPad Software, Inc.). Reliability was assessed regarding inter‐ and intraobserver correlations. Intraclass correlation coefficients (ICCs) were evaluated to determine inter‐ and intraobserver reliabilities. The criteria, based on the study by Landis and Koch [19], were as follows: nearly perfect (0.81–1.00), excellent (0.61–0.80), good (0.41–0.60) and slight (0.00–0.20). The expected ICC value ranged from 0.7 to 0.9, with statistical significance set at *p* < 0.05. Descriptive statistical analysis was conducted, and data normality was assessed using the Shapiro–Wilk test. Dependent variables were reported either as mean ± standard deviation or median with interquartile range. Statistical tests—including a paired Student's *t* test, Chi‐squared test and one‐way ANOVA, followed by a Tukey‐Kramer multiple comparison test—were applied to the dependent variables.

### Ethical approval

This study was approved by the Institutional Ethics Committee (approval number: 2021‐477, 17 November 2021). The patients and their families were informed that the study data would be submitted for publication, and their consent was obtained.

## RESULTS

**Figure 1:**
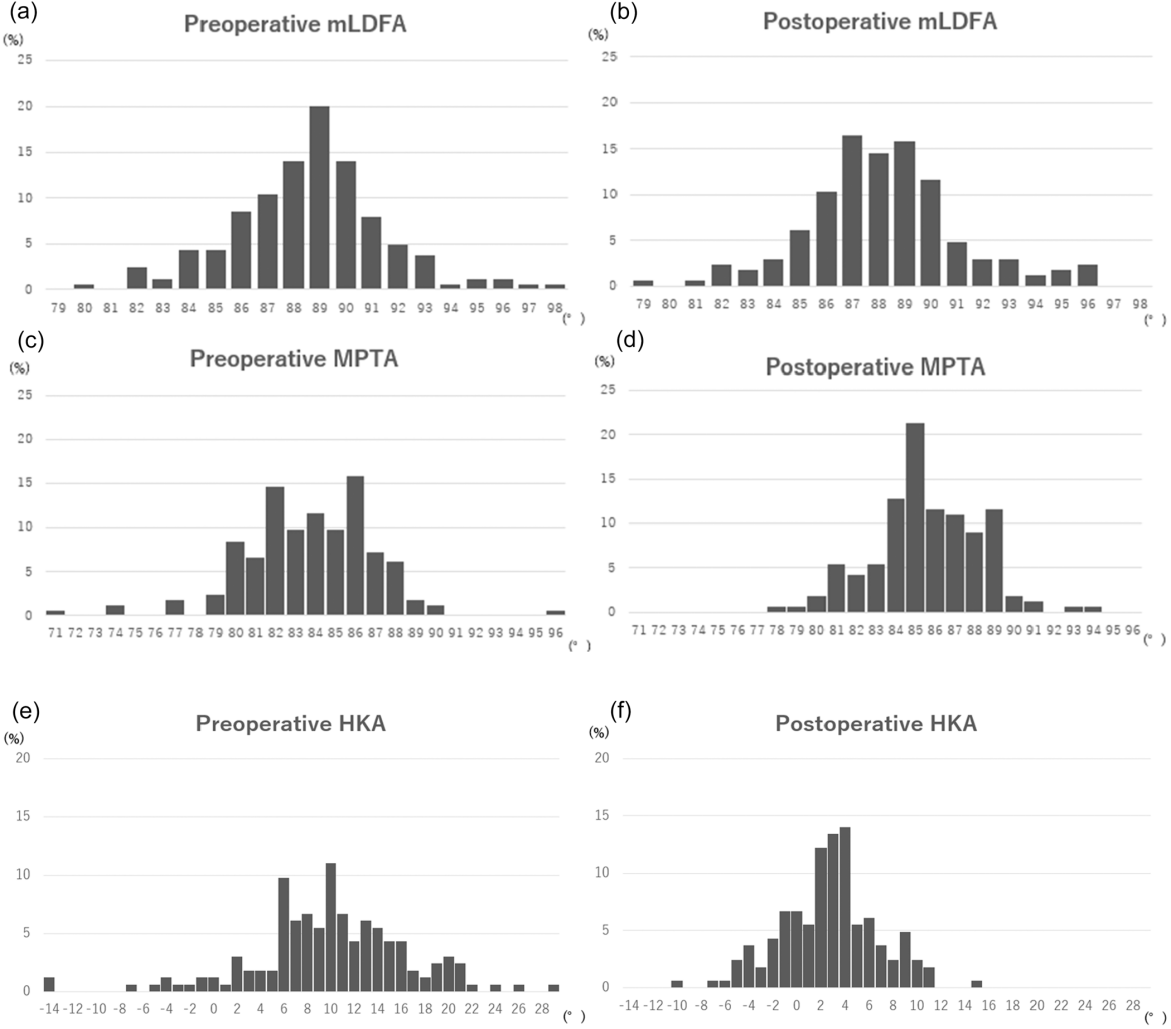
Histogram of (a) mechanical lateral distal femoral angle (mLDFA) pre‐operatively, (b) mLDFA post‐operatively, (c) medial proximal tibial angle (MPTA) pre‐operatively, (d) MPTA post‐operatively, (e) hip–knee–ankle angle (HKA) pre‐operatively and (f) HKA post‐operatively. HKA is (+) for varus and (−) for valgus.

The ICCs for reproducibility and repeatability were near perfect or excellent for the three post‐operative radiographic parameters; the preoperative and post‐operative mLDFA were 88.6 ± 2.9° (80.0–97.6°) and 88.3 ± 3.0° (79.3–96.5°), respectively, demonstrating an average deviation of 0.3° from the preoperative levels. The preoperative and post‐operative MPTAs were 83.8 ± 6.7° (71.2–96.0°) and 85.6 ± 2.6° (78.1–94.0°), respectively, demonstrating an average increase of 1.8° compared with preoperative values. The mean angle of varus relative to the tibial axis was 4.4° post‐operatively. The preoperative and post‐operative HKAs were 10.0 ± 6.6° (−14.0° to 29.0°) and 2.8 ± 3.9° (−10.0° to 15.0°), respectively, averaging 7.2° of correction compared with the preoperative values (Table 1 and Figure 1).

**Table 1:** Alignment radiological parameters.

Radiological alignment parameter	Preoperative	Post‐operative	*p* value
Mechanical lateral distal femoral angle	88.6 ± 2.9° (80.0–97.6)	88.3 ± 3.0° (79.3–96.5)	*p* = 0.27457
Medial proximal tibial angle	83.8 ± 6.7° (71.2–96.0)	85.6 ± 2.6° (78.1–94.0)	*p* < 0.01
Hip–knee–ankle angle	10.0 ± 6.6° (−14.0 to 29.0)	2.8 ± 3.9° (−10.0 to 15.0)	*p* < 0.01

*Note*: All units in degrees. Mean ± standard deviation (range).

The knee extension angles were −8.0 ± 6.8°, −0.7 ± 2.1 and −0.5 ± 1.6°, and the flexion angles were 126.4 ± 16.6°, 130.5 ± 9.5° and 130.5 ± 8.9° preoperatively, and 1 and 2 years post‐operatively, respectively (*p* < 0.01). Knee extensor muscle strength was 11.7 ± 9.4, 15.9 ± 5.5 and 15.6 ± 5.9 preoperatively, and 1 and 2 years post‐operatively, respectively, showing significant post‐operative improvement compared with preoperative values (*p* < 0.01). The gait patterns of the patients also significantly improved at 1 and 2 years post‐operatively compared with those before surgery: 69.0%, 89.0% and 89.6% for indoor independent walking; and 14.0%, 47.0% and 58.9% for single‐step stair climbing, preoperatively, and 1 and 2 years post‐operatively, respectively (*p* < 0.01). The preoperative and 1‐ and 2‐year post‐operative were KSS 13.8 ± 5.7, 26.8 ± 8.1 and 27.7 ± 8.4 for satisfaction (0–40); and 43.0 ± 16.9, 68.0 ± 19.4 and 67.2 ± 20.9 for function (0–100), respectively. The KSS satisfaction and function scores both demonstrated significant improvement from the preoperative values (*p* < 0.01); however, the FJS‐12 showed no significant difference between 1 and 2 years post‐surgery, with scores of 59.6 ± 22.1 and 63.5 ± 23.0, respectively (Table 2). There were no complications leading to revision. One case of infection was treated with debridement alone, and one case of patellofemoral disorder was alleviated by patellar replacement.

**Table 2:** Clinical evaluation.

	Preoperative	1‐Year post‐operative	2‐Year post‐operative
Extension angle (degrees)	−8.0 ± 6.8°	−0.7 ± 2.1°*	−0.5 ± 1.6°*
Flexion angle	126.4 ± 16.6°	130.5 ± 9.5°*	130.5 ± 8.9°*
Extensor muscle strength (kgf)	11.7 ± 9.4	15.9 ± 5.5*	15.6 ± 5.9*
Gait patterns			
Indoor independent walking	69.0%	89.0%**	89.6%**
Single‐step stair climbing	14.0%	47.0%**	58.9%**
Scores			
KSS satisfaction score (0–40)	13.8 ± 5.7	26.8 ± 8.1*	27.7 ± 8.4*
KSS function score (0–100)	43.0 ± 16.9	68.0 ± 19.4*	67.2 ± 20.9*
FJS‐12		59.6 ± 22.1	63.5 ± 23.0

Abbreviations: FJS‐12, Forgotten Joint Score; KSS, Knee Society Score.

* *p* < 0.01 (vs. Pre‐op) (Tukey‐Kramer multiple comparisons test)

** *p* < 0.01 (vs. Pre‐op) (chi‐square test).

## DISCUSSION

In our study, the post‐operative mLDFA was 88.3°, aligning with the results of previous studies [7, 22, 35]. Compared with the preoperative values, the mLDFA decreased by only 0.3°, suggesting that the osteotomy closely approximated the thickness of the implant. In unrestricted KA‐TKA, precise distal femoral osteotomy is critical for replicating the cylindrical axis [15]. Future research should focus on refining osteotomy techniques and exploring compensatory adjustments related to cartilage. The post‐operative MPTA and HKA were previously reported to be approximately 87.5° and 0.5°, respectively [7, 22, 35]. By contrast, our findings indicate values of 85.6° and 2.8°, leaning slightly more medially. Intriguingly, Bellemans et al. [3] documented an MPTA and HKA of 87.6° and 1.2°, respectively, in females with standard knee anatomy. Conversely, a study conducted by Wanezaki et al. in a Japanese cohort [33] reported an MPTA of 85.6° and an HKA of 2.3°, suggesting that Japanese individuals may have a smaller mLDFA and MPTA, larger HKA, and higher prevalence of constitutional varus compared to those of individuals from other countries. Moser et al. [24] have highlighted that ethnic differences in coronal plane alignment are crucial when planning TKA alignment strategies. They note that Asian populations more frequently exhibit varus and valgus phenotypes, suggesting that a neutral alignment based on MA could adversely affect knee biomechanics and functional outcomes. In contrast, KA and personalized alignment strategies, which aim to replicate a patient's natural alignment, may be particularly beneficial in Asian populations. The differences observed in post‐operative MPTA and HKA in our study, compared to other studies [7, 22, 35], may be influenced by anatomical variations associated with ethnicity, especially considering the unrestricted KA‐TKA approach employed.

This study also found significant differences between the preoperative and post‐operative measurements of both the MPTA and HKA (Table 1 and Figure 1). Regarding the MPTA, this difference was attributed to the higher incidence of cases with a preoperative MPTA < 80°. This is likely because the method used for MPTA measurement is based on points on the joint surface. In cases of knee osteoarthritis, which is common among Japanese patients, bone defects often result in an increased varus evaluation. When the tibial joint surface is osteotomized to the thickness of the implant, the preoperative and post‐operative MPTA should approximate each other. However, in such cases, the measurements tended to reflect a greater varus than the patient's original MPTA. In this study, the use of a soft‐tissue‐respecting technique, which aims to replicate the natural joint surface, was considered to have contributed to the observed differences between the preoperative and post‐operative measurements. Regarding the HKA, in addition to the influence of MPTA, the HKA in this study was measured using weight‐bearing radiographs rather than aHKA. As a result, the effect of joint line convergence angle is also believed to have contributed to the observed differences between the preoperative and post‐operative HKA measurements.

The preoperative and post‐operative knee joint extension and flexion angles, compared with other reports, are presented in Table 3. In the study by Dossett et al. [7], the average post‐operative knee extension and flexion angles for KA‐TKA were recorded as −2° and 121°, respectively, 2 years post‐operation. Their results also highlighted a notably superior flexion angle with the KA compared with the MA approach. Our own measurements revealed extension and flexion angles of −0.5° and 131° respectively, corresponding with the values reported in existing literature [7, 9, 22, 27, 31] (Table 3). Clinical outcomes, when compared with other reports, are presented in Table 4.

**Table 3:** Extension and flexion angles preoperatively and post‐operatively.

	Number of cases	Period	Extension (°)		Flexion (°)	
			Pre‐op	Post‐op	Pre‐op	Post‐op
Dossett (2014) [7]	44	2 y	4	2	117	121
Young (2017) [35]	49	2 y	3	–	118	119
McEwen (2020) [22]	41	2 y	4	0	125	127
French (2020) [9]	46	1 y	–	–	103	115
This study	164	2 y	8	0.5	126	131

**Table 4:** Results of KSS and FJS.

	Number of cases	Age	Period	KSS (Satisfaction)	KSS (Function)	FJS
Young (2017) [35]	49	72	2	–	83	69
Niki (2020) [25]	100	73	2	27.4	68.3	
Dossett (2014) [7]	44	66	2	–	77	
Luan (2020) [20]	114	66	1	25.6	66.4	–
McEwen (2020) [22]	41	65	2			79.9
French (2020) [9]	46	68	1			79.9
This study	164	75	2	27.7	67.2	63.5

Abbreviations: FJS, Forgotten Joint Score; KSS, Knee Society Score.

Previous studies have highlighted the KA method as comparable to, if not superior to, the MA method regarding patient‐centric evaluations [7, 8, 9, 20, 22, 25, 34, 35]. The findings of our study align with those of other studies [7, 9, 25, 35] regarding KSS for satisfaction and function; by contrast, our FJS‐12 findings were somewhat lower than those reported in other studies [9, 22, 35].

An important consideration that may explain this deviation is the age demographics of the patient pool. The relatively advanced age of our participants might have affected the results, potentially contributing to the observed discrepancy in the FJS‐12 values. When comparing the outcomes of surgical procedures, patient demographics (such as age) can play a pivotal role in influencing postsurgical evaluations, especially when discussing functional outcomes and joint awareness [32]. Hence, the elderly population in our study may have been more susceptible to decreased joint awareness, leading to a marginally lower FJS‐12. This hypothesis was further supported when juxtaposed with data from other studies involving potentially younger patient cohorts (Table 4).

The long‐term efficacy of KA‐TKA is often debated [14, 23, 34]. The primary concerns encompass the durability of the implants and potential complications that might emerge over prolonged post‐operative periods. The research by Howell et al. [14] thus offers an encouraging view on the endurance of KA‐TKA. Their findings revealed a noteworthy 10‐year post‐operative implant survival rate using the KA technique. Moreover, a subsequent study demonstrated that even considering tibial endoprosthesis in KA‐TKA, component migration at 2 years post‐operatively did not markedly differ from the outcomes of MA‐TKA [18].

In our study, the post‐operative HKA and mean MPTA were 2.8° and 85.6°, respectively. These values deviate from the acceptable range for surgeons who prefer the MA technique [27]. Notably, even in functional alignment approaches, a tibial varus angle exceeding 5° is often considered the upper limit; in our series, this was the mean value. Despite this deviation, it is particularly noteworthy that no complications necessitating revision surgery were observed. Still, the results of the present study should be interpreted with caution due to the relatively short follow‐up period; longer follow‐up and larger patient cohorts would be valuable for a more comprehensive understanding of the long‐term reliability of KA‐TKA. Determining the efficacy of KA‐TKA both immediately after surgery and in subsequent years would allow for a more comprehensive evaluation. Notably, the early treatment results were promising, even in a slightly more varus cohort than seen in Caucasian patients.

This study had several limitations; first, it focused on Japanese patients. While this provides valuable insights into the outcomes of KA‐TKA in the Japanese population, it may be difficult to generalize these findings to other populations with different anatomical and biomechanical characteristics [33]. Nevertheless, there have been reports suggesting that KA and other patient‐specific approaches may be beneficial in reproducing natural knee alignment, particularly in Asian patients [24]. Therefore, analyzing the Japanese population, as part of the broader Asian demographic, holds significant relevance for the understanding of KA‐TKA. Second, we only considered the outcomes within 2 years of surgery. Although short‐term consequences are crucial, a comprehensive appraisal of KA‐TKA longevity, resilience, and potential complications mandates a lengthier follow‐up. Third, the dataset originated from a solitary institution, potentially introducing biases related to surgical techniques, post‐operative care, and patient handling. Fourth, the radiographic alignment indicators were evaluated by several observers. Although interobserver and intraobserver correlations were nearly perfect or excellent, the possibility of subtle biases in the measurements could not be entirely ruled out. Fifth, the patient cohort had a mean age of 74.5 years; as indicated in the discussion section, age may have influenced some of the results, particularly regarding the FJS‐12. Finally, our discussion contrasts our findings with previous research, but lacks a directly comparable control group that underwent traditional TKA. Including such a group would have enabled a more direct evaluation of the efficacy of KA‐TKA compared with standard procedures.

## CONCLUSION

This study demonstrated that unrestricted KA‐TKA effectively achieved satisfactory post‐operative outcomes in a Japanese cohort, characterized by significant improvements in knee extension and flexion angles, muscle strength, gait patterns, and patient‐reported outcome measures (KSS satisfaction and function scores). Post‐operative MPTA and HKA alignment showed some deviations from the MA standards; however, no complications requiring revision surgery were observed, highlighting the potential of KA‐TKA to accommodate natural joint alignment. Nonetheless, this study had certain limitations, including the relatively advanced age of the patient cohort and the short follow‐up period. To validate the long‐term durability and broader applicability of these findings, further investigations involving larger and more diverse patient cohorts, as well as long‐term follow‐up and comparative studies with conventional TKA techniques, are necessary.

In conclusion, this study emphasizes the importance of personalized surgical approaches that consider individual anatomical characteristics while improving functional and clinical outcomes. The findings support the utility of KA‐TKA in the Japanese population, with promising early post‐operative results, providing a foundation for further exploration of KA‐TKA as a standard alignment strategy applicable to a wider patient demographic.

## AUTHOR CONTRIBUTIONS

Conceptualization, methodology, validation, formal analysis, data curation, writing—original draft, writing—review and editing: Takao Yamamoto. Investigation and resources: Takao Yamamoto, Shigenobu Fukushima, Shuji Toyono, Takahiro Miyaji and Takashi Ito. Supervision: Shigenobu Fukushima and Shuji Toyono. All authors contributed to the writing of the final manuscript.

## CONFLICT OF INTEREST STATEMENT

Takao Yamamoto and Shigenobu Fukushima had a speaking engagement for Medacta and delivered a presentation on kinematic alignment total knee arthroplasty. The remaining authors declare no conflicts of interest.

## ETHICS STATEMENT

This study was approved by the Institutional Ethics Committee (approval number: 2021‐477, 17 November 2021). All patients and their families provided informed consent for publication of the study data. The patients and their families were informed that the collected data would be submitted for publication, and they provided consent.

## Data Availability

The data that support the findings of this study are not publicly available due to ethical or privacy restrictions. However, they are available from the corresponding author upon reasonable request and with permission from institution and ethics committee.
